# Genistein Sensitizes Bladder Cancer Cells to HCPT Treatment In Vitro and In Vivo via ATM/NF-κB/IKK Pathway-Induced Apoptosis

**DOI:** 10.1371/journal.pone.0050175

**Published:** 2013-01-24

**Authors:** Yong Wang, He Wang, Wei Zhang, Chen Shao, Peng Xu, Chang Hong Shi, Jian Guo Shi, Yu Mei Li, Qiang Fu, Wei Xue, Yong Hua Lei, Jing Yu Gao, Juan Ying Wang, Xiao Ping Gao, Jin Qing Li, Jian Lin Yuan, Yun Tao Zhang

**Affiliations:** 1 Department of Urology, Tangdu Hospital, Fourth Military Medical University, Xi'an, Shaanxi, China; 2 Department of Urology, Xijing Hospital, Fourth Military Medical University, Xi'an, Shaanxi, China; 3 Department of Medical and Training Department, Tangdu Hospital, Fourth Military Medical University, Xi'an, Shaanxi, China; 4 Department of Experimental Animal, Fourth Military Medical University, Xi'an, Shaanxi, China; 5 Department of Cancer Research Institute, Fourth Military Medical University, Xi'an, Shaanxi, China; 6 Department of Plastic Surgery, Tangdu Hospital, Fourth Military Medical University, Xi'an, Shaanxi, China; Wayne State University School of Medicine, United States of America

## Abstract

Bladder cancer is the most common malignant urological disease in China. Hydroxycamptothecin (HCPT) is a DNA topoisomerase I inhibitor, which has been utilized in chemotherapy for bladder cancer for nearly 40 years. Previous research has demonstrated that the isoflavone, genistein, can sensitize multiple cancer cell lines to HCPT treatment, such as prostate and cervical cancer. In this study, we investigated whether genistein could sensitize bladder cancer cell lines and bladder epithelial cell BDEC cells to HCPT treatment, and investigated the possible underlying molecular mechanisms. Genistein could significantly and dose-dependently sensitize multiple bladder cancer cell lines and BDEC cells to HCPT-induced apoptosis both in vitro and in vivo. Genistein and HCPT synergistically inhibited bladder cell growth and proliferation, and induced G2/M phase cell cycle arrest and apoptosis in TCCSUP bladder cancer cell and BDEC cell. Pretreatment with genistein sensitized BDEC and bladder cancer cell lines to HCPT-induced DNA damage by the synergistic activation of ataxia telangiectasia mutated (ATM) kinase. Genistein significantly attenuated the ability of HCPT to induce activation of the anti-apoptotic NF-κB pathway both in vitro and in vivo in a bladder cancer xenograft model, and thus counteracted the anti-apoptotic effect of the NF-κB pathway. This study indicates that genistein could act as a promising non-toxic agent to improve efficacy of HCPT bladder cancer chemotherapy.

## Introduction

Bladder cancer is one of the most common malignancies affecting the urinary system. A total of 44,690 males (29.8 per 100,000) and 16,730 females (11.2 per 100,000) were diagnosed in 2006, ranking bladder cancer as the fourth commonest male and ninth commonest female malignant disease in the United States [1]. In contrast, the incidence of bladder cancer in Asia is much lower. In 2009, Zhang et al. reported that although the rates rose between 1988 and 2002 (8.22 per 100,000 in 1988–1992, 9.45 per 100,000 in 1993–1997 and 9.68 per 100,000 in 1998–2002), the incidence of bladder cancer in China remains lower than the United States [2]. Similarly, in Eastern Asia, low incidences of bladder cancer have been reported in Korea (14.39 per 100,000), Japan and India (approximately 14 per 100,000) [3]–[5]. Additionally, the 5-year disease-specific survival rates of bladder cancer patients in Asia are higher than those in Western countries [6].

The chemotherapeutic agent, hydroxycamptothecin (HCPT), is primarily used for the treatment of bladder cancer. HCPT induces apoptosis in bladder cancer cells by forming a ternary complex with DNA and the DNA enzyme topoisomerase I via hydrogen bonds, thereby stabilizing the complex. The stable complex prevents DNA re-ligation and leads to the conversion of single-strand DNA breaks into double-strand breaks during the S-phase. At this point, the replication fork collides with DNA cleavage complexes, which induces apoptosis and cell cycle arrest [7].

Genistein, a well known isoflavone and natural botanical estrogen, has been shown to inhibit cancer cell growth, survival, metastasis and angiogenesis by increasing apoptotic cell death via the induction of several DNA-damaging stimuli [8]–[10]. Genistein has been shown to have an inhibitory effect on the growth of prostate cancer [11], cervical cancer [12], breast cancer [13], colon cancer [14] and renal cell carcinoma [15] cells. Genistein can also chemosensitize many malignant tumors to the effects of DNA toxic drugs. Previous reports have indicated that pretreatment with 10–30 µmol/l genistein can chemosensitize cervical, ovarian and normal fibroblast cells to treatment with HCPT by inducing a greater degree of growth inhibition and cell apoptosis [16]. However, whether genistein can enhance the chemotherapeutic effect of HCPT in bladder cells, and its molecular mechanism of action in this tissue type, remain unclear. Therefore, we explored whether genistein could chemosensitize bladder cancer cells to HCPT, and investigated the potential underlying mechanisms of this effect.

## Materials and Methods

### 1. Cell lines

J82, SCaBER, and TCCSUP bladder cancer cell lines were purchased from the American Type Culture Collection (Manassas, VA, USA), BFTC905, HT1197, T24, TSGH-8301 bladder cancer cell lines were from the China Center for Type Culture Collection (CCTCC). The primary bladder epithelial cell line, BDEC, was from BioWhittaker (San Diego, CA, USA) and were maintained as exponentially growing cultures in DMEM supplemented with 10% fetal bovine serum, 100 U/ml penicillin and 100 µg/ml streptomycin. Genistein (Sigma, Shanghai, China) and HCPT (kindly provided by Sanofi, Shanghai, China) were dissolved in DMSO to prepare 10 mM stock solutions. For experiments, the cells were incubated for 3 days and then treated with or without 10 µM genistein and 1 µM HCPT for 24 h.

### 2. Cell growth inhibition by genistein and HCPT

Cells were seeded at a density of 5×10^3^ cells/well and allowed to attach overnight. The culture medium was replaced with fresh media containing genistein at different concentrations for 24 h, and cells were then exposed to HCPT for an additional 72 h. For each single agent treatment, the cells were treated with genistein for 96 h and HCPT for 72 h. Cell growth was examined using the MTT assay.

### 3. Flow cytometry for apoptosis

Adherent cells were trypsinized, resuspended and treated as described previously [17]. Flow cytometry was performed using blue light argon-in laser (excitation wavelength, 488 nm; laser power, 200 mW) and red fluorescence from the PI that labels DNA was recorded. All tests for apoptosis were conducted in duplicate and the results shown are representative of at least three experiments.

### 4. Immunofluorescent staining for γ-H2AX and ATM

For γ-H2AX staining, cells were treated with different concentrations of HCPT and genistein, the media was removed at various time points and the cells were fixed in 1% paraformaldehyde for 10 min followed by 70% ethanol for 10 min. The cells were then incubated in 0.1% Triton X in phosphate buffered saline (PBS) for 10 min, permeabilized in 0.5% Triton in PBS for 10 min, washed three times in PBS and blocked with 5% bovine serum albumin (BSA) in PBS for 60 min. The cells were incubated with anti–γ-H2AX (1∶2,000; Cell Signaling, Shanghai, China) or anti-ATM (1∶300, Cell Signaling, Shanghai, China) in 5% BSA in PBS at 4°C overnight, washed four times in PBS, incubated in the dark with a FITC-labeled secondary antibody (1∶2,000 for anti-γ-H2AX and 1∶300 for anti-ATM) in 5% BSA for 1 h, washed 4 times in PBS, incubated in the dark with 1 µg/ml 4′,6-diamidino-2-phenylindole (DAPI; Invitrogen, Carlsbad, CA, USA) in PBS for 5 min, and mounted and coverslipped in Fluoromount G (Southern Biotech, Birmingham, AL, USA). The slides were examined on a Leica fluorescence microscope (Wetzlar, Germany), images were captured using a Nikon fluorescence microscope (Nikon Eclipse E800) and imported into Nikon ACT-1 (Version 1.12) software. Images were combined into a publishing format using Adobe Photoshop CS2 software. For each treatment condition, the number of γ-H2AX or ATM foci was determined in at least 50 cells. All observations were validated by at least three independent experiments.

### 5. Western blotting

Bladder cancer cells were lysed in 400 µl 1% SDS lysis buffer (50 mM HEPES, pH 7.5, 100 mM NaCl, 10 mM EDTA, 4 mM NaPPi, 2 mM Na_3_VO_4_) for 5 min on ice to obtain total cell lysates. To collect nuclear protein, the monolayers were washed three times with ice-cold hypotonic lysis buffer (HLB, pH 7.5, 10 mM Tris, 10 mM KCl, 1 mM EDTA, 1 mM EGTA, 2 mM MgCl_2_), and the cells were collected and transferred to a homogenizer (Wheaton Ltd, Millville, NJ, USA) in 500 µl HLB. The cells were swollen in HLB for 15 min, homogenized and the cell nuclei were collected by centrifugation and lysed in RIPA buffer. The pull-down assay was performed by the immunoprecipitation of 500 µg nuclear or cytosolic extract (precleared with Protein A/G beads) with primary antibody, and then 50–100 µg total or nuclear lysate was resolved on a 7.5–12.5% sodium dodecyl sulfate-polyacrylamide (SDS-PAGE) gel, electro-transferred to a Hybond ECL membrane (Amersham Pharmacia Biotech, Piscataway, NJ, USA), blocked in PBS containing 5% nonfat dried milk and 0.05% Tween-20, incubated with primary antibody overnight at 4°C, and then incubated with secondary antibodies for 1 h. The protein bands were visualized using the Phototope-HRP Western Detection System (Cell Signaling, Beverly, MA, USA) and Kodar film (Perkin Elmer, Waltham, MA, USA) and scanned and quantified using ImageJ (http://rsbweb.nih.gov/ij/). The anti-ATM, anti-phospho H2AX, anti-phospho ATM (Ser 1981), anti-IKK, anti-phospho IKK1/2, anti-β-actin, anti-GAPDH, anti-phospho-NEMO (NF-kappa-β essential modulator), anti-NBS1 (Nijimegen breakage syndrome 1) antibody, anti-caspase 3 and 9, anti-phospho PARP and anti-Oct1 antibodies were obtained from Cell Signaling.

### 6. siRNA transfection

Transfection of TCCSUP and BDEC cells with ATM siRNA (50 nmol/l; Invitrogen) was performed using Oligofectamine reagent (Invitrogen) according to the manufacturer's protocol.

### 7. In vivo tumor therapy studies

The experiment was approved by the Ethics Committee of the Fourth Military Medical University. Bladder cancer cells were pre-mixed 1∶1 with Matrigel (Becton Dickinson, Beijing, China) and subcutaneously inoculated (5×10^6^ cells per site) into the flanks of 10-week-old female SCID nude mice (Department of Experimental Animals, the Fourth Military Medical University, Xi'an, Shaan'xi, China). Drug treatment started 22 days after tumor cell injection. Mice were randomly divided (5 mice/group) into five groups. The control group was compromised tumors that were treated with 0.01% DMSO. Mice were treated orally with food that contained genistein (1 g/kg) and/or the transperitoneal injection of 3 µg/ml HCPT. The percentage change in tumor size was calculated by comparisons with the baseline value at 22 days.

### 8. Electrophoretic mobility shift assay (EMSA)

Nuclear cell extracts were obtained as previously described, and 10 µg nuclear extract was incubated with purified ^32^P-labeled NF-κB consensus double-stranded oligonucleotide and 0.25 mg/mL poly(dI-dC) in 5× binding buffer [18]. Samples were separated on 8% polyacrylamide gels, the gels were dried, exposed to X-ray film overnight at −80°C and developed using an All-Pro 100 Plus automated X-ray film processor (All-Pro Imaging Corporation, Hicksville, NY, USA).

### 9. Quantification and statistical methods

Groups were compared using the Student's *t*-test; *P* values≤0.05 were considered statistically significant.

## Results

### 1. Effects of HCPT and genistein on the viability of bladder cancer cells

**Figure 1:**
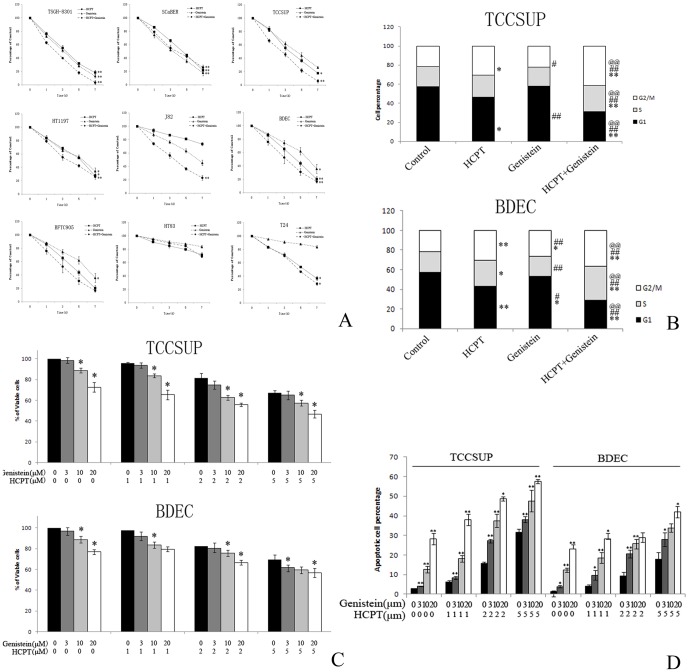
Genistein sensitizes bladder cancer cells to HCPT treatment. **A.** MTT assay of bladder cancer cell lines and BDEC cells treated with 10 µM genistein and/or 1 µM HCPT; results are expressed as percentage of control cells. **B**. MTT assay of TCCSUP and BDEC cells treated with different concentrations of HCPT and/or genistein for 48 h. **C.** Cell cycle distribution of TCCSUP or BDEC treated with 1 µM HCPT and/or 10 µM genistein for 24 h. **D.** Apoptosis measured by FACS in TCCSUP, and BDEC treated with 10 µM genistein and/or 1 µM HCPT. Values are mean ± SEM of three independent experiments; *P<0.05 and **P<0.01 compared with control group. #*P*<0.05 and ##*P*<0.01 compared with HCPT; @*P*<0.05 and @@P<0.01 compared with genistein.

Bladder cells were treated with genistein for 3 days, and cell viability was determined using the MTT assay. The treatment of a variety of bladder cancer cell lines and BDEC bladder cells with genistein resulted in the dose- and time-dependent inhibition of cell proliferation, which demonstrated that genistein reproducibly inhibits the growth of bladder cancer cells. However, a synergistic effect was observed when J82, T24, TSGH8301 and TCCSUP bladder cancer cells and BDEC bladder cells were treated with different concentrations of genistein and HCPT (Fig. 1A). MTT assays indicated that the synergistic effect of genistein and HCPT was concentration-dependent (Fig. 1B).

### 2. Synergistic cell cycle arrest by HCPT and genistein

Both HCPT (1 µm) and genistein (10 µm) for a 24-hour period showed a statistically significant ability to arrest the cell cycle (Fig. 1C). Compared with the vehicle, HCPT caused a cell cycle arrest in both the S phase (control: 57.6%, HCPT: 46.5%, genistein: 57.9%, HCPT+genistein: 31.1% for TCCSUP cells; control: 57.2%, HCPT: 43.1%, genistein: 53.6%, HCPT+genistein: 29.1% for BDEC cells) and the G2-M phase (control: 21.5%, HCPT: 30.6%, genistein: 22.1%, HCPT+genistein: 41.6% for TCCSUP cells; control: 21.7%, HCPT: 30.3%, genistein: 26.7%, HCPT+genistein: 36.3% for BDEC cells). Although genistein (10 µm) alone did not affect the cell cycle significantly compared with the controls, the addition of genistein to HCPT-treated (1 µm) cells significantly sensitized the cells to HCPT via inducing G2/M cell cycle arrest.

### 3. Synergistic induction of apoptosis by HCPT and genistein

Using FACS to quantify apoptosis, we observed that 10 µM genistein and 1 µM HCPT synergistically and dose-dependently induced apoptosis in bladder cancer cells (Fig. 1D). The induction of apoptosis was dose-dependent and directly correlated with an inhibition of cell growth (Fig. 1B, D).

### 4. NBS1-dependent ATM activation is induced by DNA damage

**Figure 2:**
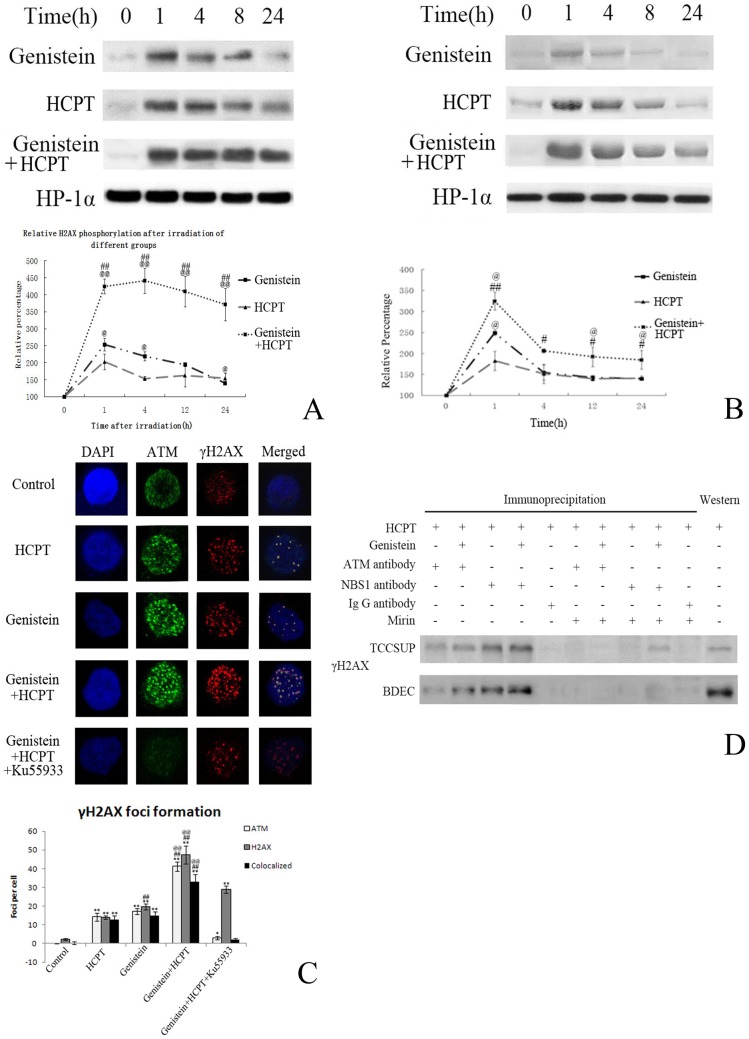
Genistein augments HCPT-induced cell DNA damage via the synergistic activation of nuclear ATM. **A**. Western blot and quantification of H2AX DNA damage repair after the pretreatment of 10 µM genistein and/or 10 µM HCPT. HP-1α was used as a loading control; results are expressed relative to the control group at 0 h. **B**. Western blot and quantification of ATM Ser 1981 phosphorylation after the pretreatment of TCCSUP cells with 10 µM genistein and/or 10 µM HCPT for 1 h. **C**. Representative images and quantification of ATM phosphorylation and H2AX foci formation 30 min after treatment of TCCSUP cells with 1 µM HCPT and/or 10 µM genistein; discrete foci of ATM autophosphorylation appear at putative sites of double strand breaks. **D**. Identification of the NBS1-dependent ATM/H2AX interaction. TCCSUP cells and BDEC cells were treated with 1 µM HCPT and/or 10 µM genistein for 1 h with or without the NBS1 inhibitor, mirin, immunoprecipitated with anti-ATM or anti-NBS1 antibodies and immunocomplexes were detected by Western blotting. ATM foci increased linearly with dose after 1 h genistein and HCPT treatment. Values are mean ± SEM of three independent experiments; IP: immunoprecipitation, W: Western-blot. *P<0.05 and **P<0.01 compared with control group. #*P*<0.05 and ##*P*<0.01 compared with HCPT; @*P*<0.05 and @@P<0.01 compared with genistein.

The results described in section 3.3 indicated that genistein may act synergistically with HCPT to induce apoptosis. As stated above, HCPT could induce cell apoptosis via inducing double strand breaks (DSB) in DNA. Therefore, we investigated whether this synergism of apoptosis is related to DSB. TCCSUP cells were treated with 1 µM HCPT and/or 10 µM genistein for 1 h, and the total protein from each group was extracted and underwent Western blotting for the chromosomal histone protein, γ-H2AX [19]. The Western blot demonstrated that HCPT and genistein could synergistically induce H2AX phosphorylation at 1 hour after drug treatment, which indicated that these drugs could induce DSB. Furthermore, strong H2AX phosphorylation could still be seen in the co-treated group 24 h after treatment compared with the cells administered with single drugs only, which demonstrated a delayed DNA damage repair process (Fig. 2A). Additionally, 1 µM HCPT and 10 µM genistein synergistically activated the phosphorylation of ATM at Ser 794 in a dose-dependent manner (Fig. 2B). The spatial distribution of ATM and γ-H2AX after drug treatment was determined using a multiplexed immunofluorescence assay (Fig. 2C). Significantly more ATM and γ-H2AX nuclear foci were observed in TCCSUP cells treated with both HCPT and genistein for 30 min compared to the control, HCPT- and genistein-treated cells. Additionally, treatment with both these drugs significantly increased the co-localization of ATM and γ-H2AX in the cell nucleus. The ATM inhibitor, Ku55933, significantly decreased ATM foci formation, and thereby inhibited γ-H2AX/ATM co-localization in HCPT- and genistein-treated cells (Fig. 2C). An immunoprecipitation assay was performed to explore how ATM is phosphorylated after HCPT and genistein treatment. Treatment with both HCPT and genistein activated ATM and induced an interaction between ATM/H2AX and NBS1/H2AX in TCCSUP and BDEC cells. The NBS1 inhibitor, mirin, significantly attenuated HCPT- and/or genistein-induced ATM or NBS1 and H2AX binding in HCPT- and genistein-treated cells (Fig. 2D).

### 5. ATM inhibition downregulates NF-kB and induces apoptosis in HCPT- and genistein-treated cells

**Figure 3:**
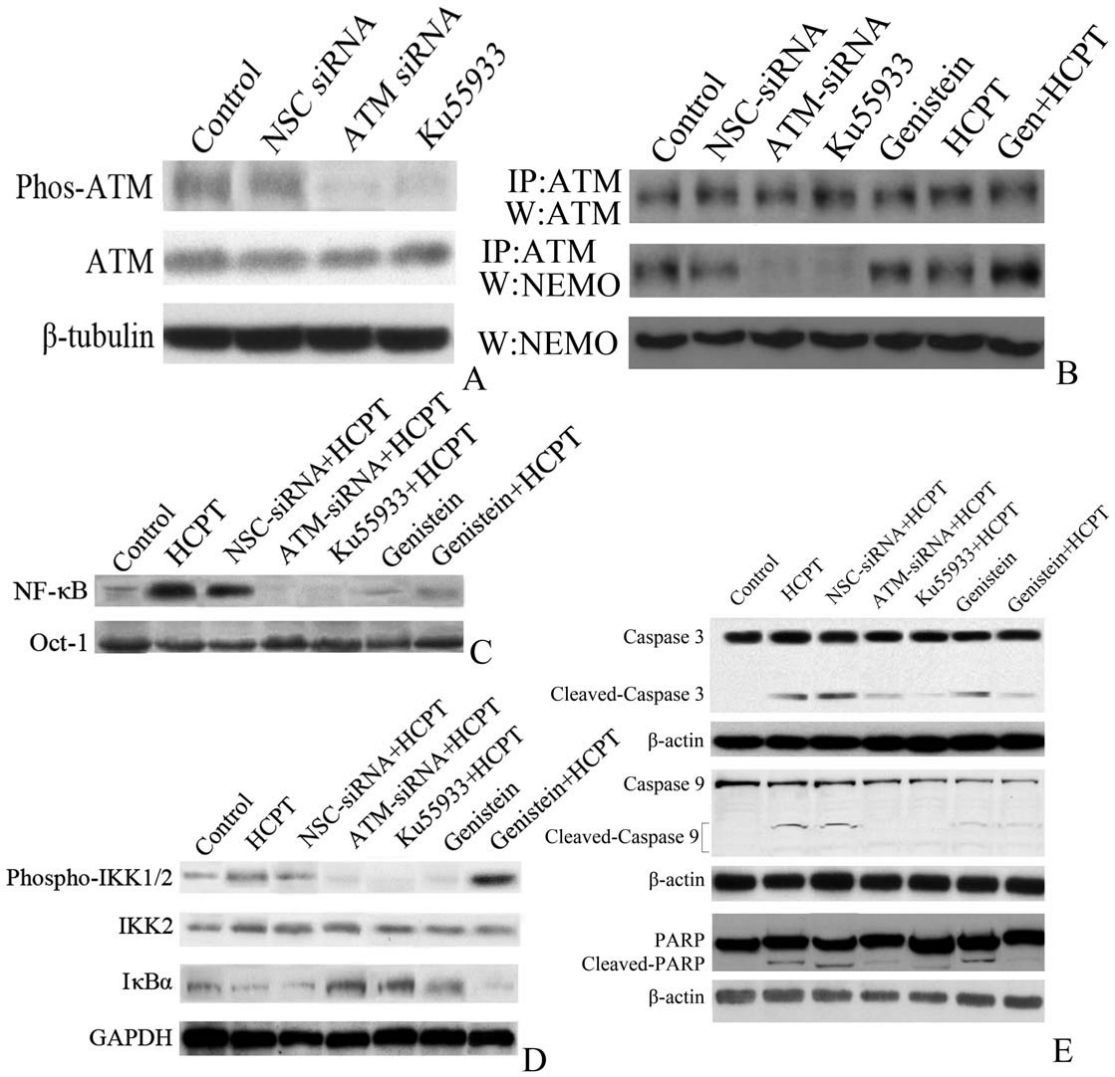
Genistein attenuates the activation of the ATM/NF-κB pathway by HCPT. **A.** Western blot of ATM phosphorylation in TCCSUP cells treated with 1 µM HCPT for 1 h transfected with non-silencing control (NSC) siRNA, ATM siRNA or treated with the ATM inhibitor, Ku55933 (10 µM,2 h). **B**. Immunoprecipitation and Western blot of ATM and NEMO in TCCSUP cells transfected with NSC siRNA or ATM siRNA, or treated with 10 µM Ku55933, 10 µM genistein and/or 1 µM HCPT. IP: immunoprecipitation, W: Western-blot. **C.** EMSA blot of NF-κB expression in TCCSUP cells treated with 10 µM genistein and/or 1 µM HCPT in the presence of NSC siRNA and ATM siRNA. **D**. Western blot of NF-κB, IKK2, IκBα expression and IKK1/2 phosphorylation in TCCSUP cells treated with 10 µM genistein and/or 1 µM HCPT in the presence of NSC siRNA and ATM siRNA, indicating that genistein and HCPT induce the phosphorylation of IKK1/2 and increase IκBα expression via ATM. **E**. Western blot of PARP, caspase 3 and caspase 9 cleavage in whole cell lysates prepared from TCCSUP cells treated with 10 µm genistein and/or 1 µM HCPT in the presence of ATM siRNA or Ku55933. All experiments were repeated three times, and similar results were obtained in each replicate.

The levels of activated, phosphorylated ATM were investigated in TCCSUP cells. TCCSUP cells treated with 1 µM HCPT for 1 h exhibited a strong constitutive ATM phosphorylation, which could be inhibited by ATM siRNA or the small molecular specific ATM inhibitor, KU55933 (Fig. 3A). Immunoprecipitation assays indicated that ATM siRNA reduced ATM/NEMO binding in TCCSUP cells; however, HCPT and genistein could synergistically augment ATM/NEMO binding (Fig. 3B), which indicated that NEMO plays an important role in suppression of HCPT induced NF-κB activation. Additionally, the synergistic augmentation of ATM/NEMO binding in HCPT- and genistein-treated cells was accompanied by increased IκBα expression (Fig. 3C) and reduced IKK1/2 phosphorylation (Fig. 3D); in turn, these induced the increased cleavage of caspase 3, caspase 9 and PARP (Fig. 3E).

### 6. Genistein attenuated HCPT-induced NF-κB-activation and thus synergistically induced apoptosis in vivo

**Figure 4:**
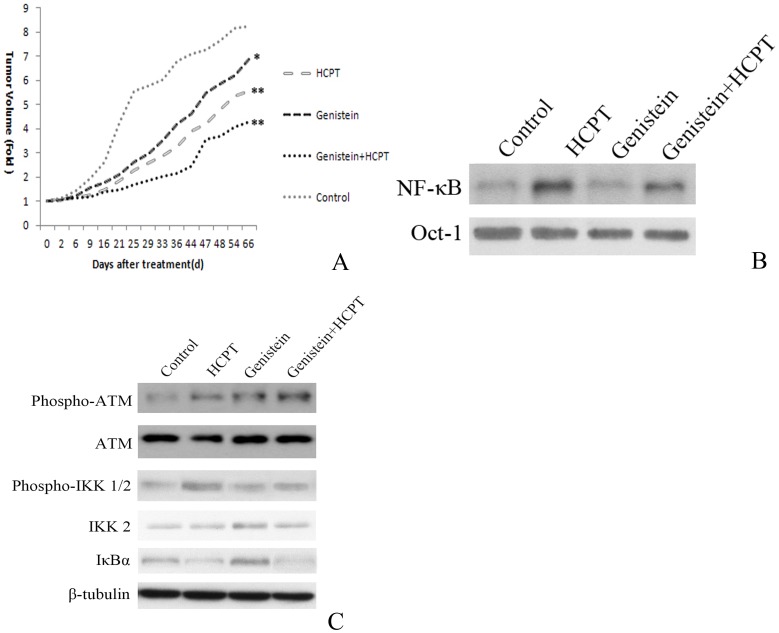
Genistein sensitizes TCCSUP tumors to HCPT in a SCID xenograft model. TCCSU bladder cancer cell tumors were allowed to establish for 22 days, then the animals were injected intratumorally with 10 µM genistein and/or 10 µM HCPT on day 0 and 7 of treatment. **A**. Growth curve of TCCSUP bladder cancer xenografts treated with genistein and/or HCPT; tumor volume is expressed relative to tumor size at the start of treatment. **B**. EMSA assay of NF-κB expression in xenograft tumor tissues from each group. **C**. Western blot analysis of phosphorylated IKK1/2, IKK2, IκBα expression in xenograft tumor tissues from each group.

To verify the synergistic inhibitory effect on growth by genistein and HCPT in bladder cancer cells, we determined their effects in a xenograft model of SCID mice treated with the TCCSUP bladder cancer cell line. Genistein and HCPT exhibited a synergistic inhibitory effect on tumor growth in the xenograft (Fig. 4A). Furthermore, tumors treated with HCPT showed NF-κB activation, while genistein attenuated this activation (Fig. 4B). Decreased activation of the downstream molecules IKK1/2, increased phosphorylation of IκBα and increased cleavage of caspase 3, caspase 9 and PARP were observed in tumors treated with genistein and HCPT (Fig. 4C).

## Discussion

Genistein is regarded to be a potentially ideal chemotherapy agent for bladder cancer, as it is natural, safe, with minimal side effects and relatively low costs [20]. Previous investigations have indicated that soybean isoflavone, which is present in large amounts of soybean products, may play an important role in the inhibition of tumorigenesis [21]. It has also been shown to induce cell apoptosis via decreasing the expression of the 32 kDa caspase 3 precursor and increasing the levels of the cleaved active form of this caspase [22]. Zhou et al. reported that soybean isoflavones and soy phytochemical concentrates could inhibit the growth of murine and human bladder cell lines in vitro and in vivo in a dose-dependent manner [23]. Previous investigations revealed that the synergistic inhibitory effect of genistein and camptothecin in cervical cancer, ovarian carcinoma and mouse fibroblast cells resulted from their inhibition of NF-κB translocation and the induction of G2/M cell cycle arrest and apoptosis [16].

Whether genistein could sensitize bladder cells to camptothecin treatment has not been studied previously. In this study, genistein and HCPT were found to inhibit the growth of multiple bladder cancer cell lines and the primary BDEC bladder epithelial cell line (Fig. 1A). Genistein and HCPT synergistically and dose-dependently inhibited cell survival (Fig. 1B), induced G2/M cell cycle arrest (Fig. 1C) and apoptosis (Fig. 1D) in the TCCSUP bladder cancer cell line and BDEC bladder epithelial cells. With regards to the underlying mechanism, the induction of dose-dependent synergistic DNA damage by genistein and HCPT and their inhibitory effect on the DNA damage repair process was observed for up to 24 h, compared with genistein or HCPT treatment alone (Fig. 2A). As previously reported, both HCPT [24] and genistein [25] could directly inhibit DNA topoisomerase I. We hypothesize that the induction of DNA damage by HCPT and genistein is due to their inhibition on the DNA topoisomerase and thus their effects on the formation of the replication fork, which requires further investigation.

Then, we investigated the downstream signaling effects of genistein- and HCPT-induced DNA damage to determine how the synergistic induction of ATM phosphorylation is related to their pro-apoptotic effect. ATM kinase is a key regulator that is activated by DNA damage [26]. It has been reported to be closely correlated with cell apoptosis in multiple cancer cells. Zuco et al. reported that the camptothecin derivative, ST1968, can induce apoptosis via the activation of ATM [27]. Kawakami et al. reported that doxorubicin can induce apoptosis in A549 lung adenocarcinoma cells by ATM activation [28]. In this study, it was found that a combined treatment with genistein and HCPT synergistically induced ATM Ser 1981 phosphorylation (Fig. 2B) at sites of DNA damage. In cells treated with genistein and HCPT, the inhibition of ATM by its specific inhibitor, Ku55933, inhibited THE phosphorylation of ATM and H2AX, and thus inhibited their co-localization (Fig. 2C).

NBS1 has been proven to be the key element of the MRE11/RAD50/NBS1 complex, which forms immediately after a DNA DSB forms to recruit related proteins to repair the damaged DNA sites [29]. ATM and NBS1 both bind to H2AX, a scaffold protein at sites of DNA damage. As previously described [30], we found that the ability of genistein and HCPT to synergistically induce DNA damage in bladder cancer cells via ATM activation was dependent on NBS1, as the NBS1 inhibitor mirin specifically abrogated HCPT- and genistein-induced ATM/H2AX binding and NBS1/H2AX binding (Fig. 2D). These findings indicated that the synergistic DNA damaging effect of these two drugs was due to their inhibition of ATM, which is NBS1-dependent. The phosphorylation of ATM could activate NF-κB pathway via NEMO [31], which leads to the activation and expression of a variety of pro-proliferative and anti-apoptotic genes, thus protecting cancer cells from apoptosis. NEMO is thought to be a polyubiquitin binding subunit, which recruits IKK to linear or K63-linked polyubiquitin scaffolds that form as a consequence of receptor-initiated signaling events [32]. Therefore, after treatment with DNA toxic drugs, such as HCPT, DSB induced ATM activation, and the downstream NF-κB pathway is activated via NEMO (Fig. 3B). However, the NF-κB pathway has been shown to protect cells from cell apoptosis, which could in part attenuate the toxic effects of HCPT. In our research, genistein treatment could inactivate the NF-κB pathway in bladder cancer and epithelial cells. In summary, upon HCPT treatment, DNA damage may induce ATM phosphorylation, which activates the NF-κB pathway to protect cells from apoptosis. However, the multiple protease ability of genistein helps to abrogate HCPT-induced NF-κB activation [33]. The knockdown of ATM completely blocked the ability of HCPT and genistein to induce the activation of NEMO/IKK (Fig. 3D) and inhibited the cleavage of caspase 3, caspase 9 and PARP (Fig. 3E). This indicated that ATM plays a central role in HCPT- and genistein-induced apoptosis.

To confirm the synergistic effects of HCPT and genistein, we performed in vivo xenograft experiments. In xenografts of TCCSUP cells grown in SCID mice, combined treatment with HCTP and genistein synergistically inhibited tumor growth (Fig. 4A). This intracellular molecular event is in accordance with previously discovered findings in bladder cells. HCPT was shown to activate NF-κB, which was counteracted by genistein in SCID mice (Fig. 4B). Co-treatment with these two drugs synergistically activated ATM and inhibited IκBα (Fig. 4C).

This research indicates that the isoflavone, genistein, can significantly strengthen the effects of the bladder cancer chemotherapy agent, HCPT, both in vivo and in vivo. The synergistic pro-apoptotic effects of these two drugs induce more DSBs and delay the DNA damage repair process by activating the ATM/NBS1/NEMO/IKK pathway. However, some aspects of this mechanism remain to be elucidated. Firstly, it remains unknown as to whether the synergistic DSB inducing effect of HCPT and genistein occurs via interference with the replication fork and toposoimerase I. Secondly, it is still unclear how the DSB repair process is delayed, whether this is through the inhibition of homologous recombination, or the inhibition of non-homologous end joining. Thirdly, the role of the synergistic inhibition of NBS-1 activation by HCPT and genistein in the malformation of the MRN complex requires further exploration. In the end, genistein is a well-known botanic estrogen, and estrogen has been shown to be expressed in bladder transitional cell cancer, and it was negatively correlated with tumor grade [34]. Whether the estrogen effect of genistein is correlated with the synergistic growth inhibitory effect remain to be explored in the future.

**Figure 5:**
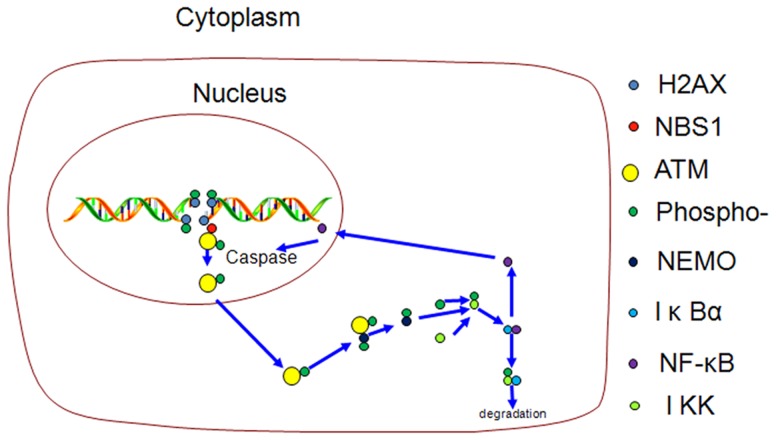
Schematic illustration of ATM activation and cytoplasmic translocation. ATM is phosphorylated at sites of double strand DNA breaks by NBS1 in the presence of H2AX phosphorylation. Activated ATM is transported to the cytoplasm, and activates NEMO, which phosphorylates IKK1/2. IKK1/2 activates and ubiquitinizes IκBα, leading to IκBα degradation. This stimulates the release and transport of NF-κB to the nucleus, where it binds to DNA, activates caspase cleavage and initiates apoptosis.

In conclusion, this study demonstrates that genistein can sensitize bladder cancer cell lines to HCTP, leading to a synergistic dose-dependent inhibition of proliferation and an induction of cell cycle arrest and apoptosis. Genistein and HCTP induce double-stranded DNA breaks, which leads to the synergistic activation of ATM, attenuates NEMO/NF-κB/IKK/caspase signal transduction, and thus induces apoptosis both in vitro and in vivo. Genistein could also counteract HCPT-induced NF-κB pathway activation, and thus attenuate the anti-apoptotic effect of the NF-κB pathway, as summarized in Fig. 5. These findings indicate that, although the underlying mechanisms require further exploration, the combined administration of HCTP and genistein might be a promising approach for the treatment of human bladder cancer.
